# Erythropoietin is a JAK2 and ERK1/2 effector that can promote renal tumor cell proliferation under hypoxic conditions

**DOI:** 10.1186/1756-8722-6-65

**Published:** 2013-09-03

**Authors:** Makito Miyake, Steve Goodison, Adrienne Lawton, Ge Zhang, Evan Gomes-Giacoia, Charles J Rosser

**Affiliations:** 1 Cancer Research Institute, MD Anderson Cancer Center Orlando, Orlando Florida, USA; 2 Nonagen Bioscience Corp, Orlando, Florida, USA; 3 Department of Pathology, Orlando Health, Orlando, Florida, USA; 4 University of Central Florida, College of Medicine, 6900 Lake Nona Blvd, Orlando, FL 32827, USA

**Keywords:** Cancer, ERK1/2, Erythropoietin, Hypoxia, JAK2, Renal

## Abstract

**Background:**

Erythropoietin (EPO) provides an alternative to transfusion for increasing red blood cell mass and treating anemia in cancer patients. However, recent studies have reported increased adverse events and/or reduced survival in patients receiving both EPO and chemotherapy, potentially related to EPO-induced cancer progression. Additional preclinical studies that elucidate the possible mechanism underlying EPO cellular growth stimulation are needed.

**Methods:**

Using commercial tissue microarray (TMA) of a variety of cancers and benign tissues, EPO and EPO receptor immunohistochemical staining was performed. Furthermore using a panel of human renal cells (Caki-1, 786-O, 769-P, RPTEC), *in vitro* and *in vivo* experiments were performed with the addition of EPO in normoxic and hypoxic states to note phenotypic and genotypic changes.

**Results:**

EPO expression score was significantly elevated in lung cancer and lymphoma (compared to benign tissues), while EPOR expression score was significantly elevated in lymphoma, thyroid, uterine, lung and prostate cancers (compared to benign tissues). EPO and EPOR expression scores in RCC and benign renal tissue were not significantly different. Experimentally, we show that exposure of human renal cells to recombinant EPO (rhEPO) induces cellular proliferation, which we report for the first time, is further enhanced in a hypoxic state. Mechanistic investigations revealed that EPO stimulates the expression of cyclin D1 while inhibiting the expression of p21^cip1^ and p27^kip1^ through the phosphorylation of JAK2 and ERK1/2, leading to a more rapid progression through the cell cycle. We also demonstrate an increase in the growth of renal cell carcinoma xenograft tumors when systemic rhEPO is administered.

**Conclusions:**

In summary, we elucidated a previously unidentified mechanism by which EPO administration regulates progression through the cell cycle, and show that EPO effects are significantly enhanced under hypoxic conditions.

## Background

Tumor cells within a growing lesion often need to adapt and survive in hypoxic conditions. One-way tumor cells are known to respond to hypoxia is to up-regulate the transcription factor hypoxia inducible factor (HIF). HIF has two subunits, HIF-1α and HIF-1β [1], and intracellular oxygen levels can modulate HIF-1α levels, while HIF-1β is constitutively expressed [2]. In normoxic conditions, it has been shown that a complex including functional von Hippel-Lindau (pVHL), a key tumor suppressor gene in clear cell renal cell carcinoma (RCC) is able to rapidly degrade HIF-1α [3]. However, in the absence of a functional pVHL, HIF-1α can accumulate, in hypoxic or normoxic conditions [4,5]. When the HIF complex translocates to the nucleus it binds to hypoxia-response elements of DNA leading to the regulation of multiple hypoxia-inducible genes [6,7]. One of the lesser-known hypoxia-inducible genes encodes the glycoprotein, erythropoietin (EPO), which is in fact a hormone, produced by the kidneys and to a lesser extent the liver [8]. EPO stimulates the production of red blood cells in the bone marrow [9]. Accordingly, one of the key indications for its use is in the management of severe anemia [10], a situation that can often occur during the administration of cytotoxic chemotherapy in the treatment of malignancies.

Recently, concerns have arisen over the potential of recombinant human erythropoietin (rhEPO) treatment and an association with tumor growth [11,12]. The effect may be induced through interaction with tumor cell EPO receptors (EPOR), which when activated promote the tumor vascularization required for adequate oxygenation [13,14]. An understanding of the mechanism of EPO in tumor biology and when EPO treatment is likely to be efficacious is an important goal at this juncture. In this study, we performed a series of *in vitro* and *in vivo* analyses to test whether EPO can stimulate the growth of renal cells. We found that rhEPO administration stimulated cellular proliferation, and the effect was enhanced in a hypoxic state, which we report for the first time. Mechanistic investigations revealed that EPO stimulates the expression of cyclin D1 while inhibiting the expression of p21^cip1^ and p27^kip1^ through the phosphorylation of JAK2 (JAK-Stat pathway) and ERK1/2 (MAPK pathway), leading to a more rapid progression through the cell cycle. We were also able to demonstrate that the growth of renal cell carcinoma xenograft tumors was increased in tumors with increased hypoxia when systemic rhEPO was administered. These investigations provide some insight into the mechanism of EPO in tumor cell stimulus, and show that the effects are significantly enhanced in association with hypoxic conditions.

## Materials and method

### Immunohistochemistry

Commercial tissue microarrays (TMA) (MC5003a, US Biomax, Inc., Rockville, MD) constructed from clinical samples obtained from a cohort of 500 patients (400 malignant tissues and 100 benign tissues from 20 different organs) were examined by immunohistochemical staining. The clinicopathologic variables of the study cohort are available at http://www.biomax.us/tissue-arrays/Multiple_Organ/MC5003a. TMAs were examined by H&E for histological verification of disease status. TMAs were deparaffinized followed by antigen retrieval using citric acid buffer (pH 6.0, 95°C for 20 mins). Slides were treated with 1% hydrogen peroxide in methanol to block endogenous peroxidase activity. After 20 mins of blocking in 1% bovine serum albumin (BSA), the TMAs were incubated overnight at 4°C with anti-human EPO antibody (sc-7956; rabbit polyclonal, dilution 1/200 in 1% BSA) and anti-human EPOR antibody (sc-695; rabbit polyclonal, dilution 1/100 in 1% BSA) from Santa Cruz Biotechnology (Santa Cruz, CA). Next, the slides were incubated with 2 μg/mL of biotinylated anti-rabbit IgG secondary antibody (Vector Laboratories, Burlingame, CA) for 30 mins at room temperature. Subsequently, the sections were stained using Standard Ultra-Sensitive ABC Peroxidase Staining kit (Pierce/Thermo Fisher Scientific, San Jose, CA) and 3, 3'- diaminobenzidine (DAB; Vector Laboratories), counterstained by hematoxyline, dehydrated, and mounted with a cover slide. Mouse xenograft tumors from the human renal cancer cell line Caki-1, known to stain strongly for EPO and EPOR were used as a positive control.

The proportion of positive cells was scored by two investigators (AL, MM) in four grades and represented the estimated proportion of immunoreactive cells (0 = 0% of cells; 1 = 1% to 40%; 2 = 41% to 75% and 3 = 76% to 100%). The intensity was scored and represented the average intensity of immunopositive cells (0 = none; 1 = weak; 2 = intermediate and 3 = strong). The proportion and intensity scores were combined to obtain a total EPO or EPOR staining score, which ranged from 0 to 6. The EPO or EPOR expression level was determined based on the total EPO or EPOR staining score as follows: none = 0, low = 1 or 2, moderate = 3 or 4, high = 5 or 6 [15]. A third investigator (CJR) reviewed discrepancies and rendered a final score. The comparison between EPO and EPOR expression in human tumors and benign tissues was calculated using Mann–Whitney U test.

### Cells, reagents and equipment

Human renal cancer cell lines; Caki-1, 786-O, 769-P (ATCC, Manassas, VA), and the normal primary human renal tubule epithelial cells (RPTEC; Lonza, Walkersville, MD) were available for analysis. Cancer cell lines were maintained in RPMI1640 medium supplemented with 10% fetal bovine serum, 50 units/ml penicillin and 50 mg/ml streptomycin (Invitrogen Corporation, Carlsbad, CA). RPTEC was maintained in renal epithelial cell basal medium (REBM) supplemented with REGM complex (Lonza CC-3190). All cells were incubated in humidified atmosphere at 37°C in air with 5% CO_2_ (normoxic conditions). For hypoxic conditions, cells were incubated at 37°C containing 1% O_2_, 5% CO_2_, and balance N_2_ in a humidified incubator. The oxygen level was automatically maintained with an oxygen controller (ProOx P110; Biospherix, Redfield, NY) supplied with compressed nitrogen gas. Recombinant human EPO (rhEPO) was purchased from R&D Systems, Inc. (Minneapolis, MN).

### Immunoblotting

Whole cell lysates were prepared using RIPA buffer with Halt Protease Inhibitor Cocktail (Thermo Fisher Scientific) as previously reported [16]. Twenty micrograms of total protein (assessed using BCA protein assay) were subjected to SDS-PAGE using Mini-PROTEAN TGX precast gels (Bio-Rad Laboratories, Richmond, CA). Proteins were transferred to polyvinylidene difluoride (PVDF) membrane (Bio-Rad). Anti-human pVHL (#2738, dilution 1:1 000), HIF-2α (#7096, dilution 1:1 000), p-Jak2 (#4406, dilution 1:1 000), total Jak2 (#3230, dilution 1:1 000), p-Stat5 (#9359, dilution 1:1 000), total Stat5 (#9363, dilution 1:1 000), p-Akt (#4060, dilution 1:1 000), total Akt (#9272, dilution 1:1 000), p-ERK1/2 (#4370, dilution 1:1 000), total ERK1/2 (#9102, dilution 1:1 000), cyclin D1 (#2978, dilution 1:1000), cyclin D3 (#2936, dilution 1:1 000), CDK4 (#2906, dilution 1:1 000), CDK6 (#3136, dilution 1:1 000), p21^cip1^ (#2947, dilution 1:1000), p27^kip1^ (#3686, dilution 1:1 000) and p15 (#4822, dilution 1:1 000) were purchased from Cell Signaling Technology. Anti-human HIF-1α (sc-53546, dilution 1:200), VEGF (sc-152, dilution 1:200), EPO (sc-7956, dilution 1:1 000), total EPOR (sc-697, dilution 1:1 000) and p-EPOR (sc-20236, dilution 1:1 000) antibodies were purchased from Santa Cruz Biotechnology. Equal loading was confirmed with β-actin (AC-15, dilution 1:10 000, Sigma-Aldrich) [17]. Stained proteins were detected using the ECL Plus Western Blotting Detection System (GE Healthcare).

### Proliferation and viability assay

Human renal cells Caki-1, 786-O, 769-P and RPTEC were plated in 96 well dishes in triplicate (10^3^ cells/well) and incubated in normoxic condition. Cells were then subjected to increasing doses of rhEPO (0–50 units/mL) and incubated in normoxic or hypoxic conditions. After 48 hrs, cell proliferation was determined by CellTiter-Glo Luminescent cell viability assay (Promega, Madison, WI) according to manufacturer’s instructions. Luminescence was measured using a FLUOstar Optima Reader (BMG LABTECH, Ortenberg, Germany). Three independent experiments were performed in triplicate.

### Cell cycle analysis

Human renal cells were seeded in 6-well plates at a density of 2 × 10^5^ cells per well and incubated for 24 hrs. Cells were starved for 18 hrs in serum/growth factors-free media containing 0.1% BSA in normoxic or hypoxic condition. After starvation, media were replaced with fresh media containing 2% FBS with or without 2 units/mL of rhEPO and incubated for 10 hrs in normoxic or hypoxic condition. Cells were harvested and fixed with 70% ethanol overnight at -20°C. Next, cells were suspended in propidium iodide (PI) staining buffer containing 50 μg/ml PI and 200 μg/ml RNase A and incubated in 37°C for 15 min. PI fluorescence was determined by flow cytometry using a FACSCalibur and CellQuest software for acquisition (BD Biosciences, San Jose, California). Cell cycle phase distribution was analyzed and reported by using FlowJo software (TreeStar Inc., Ashland, OR). Three independent experiments were performed in triplicate.

### Cell synchronization and measurement of DNA synthesis using EdU labeling

To obtain populations of cells in G_0_/G_1_ phase, all human renal cells were arrested by double thymidine block as described previously [18]. Briefly, human renal cells were seeded at 5 × 10^4^ cells per well in a 6-well plate. Cells were blocked for 18 hrs with 2.5 mM thymidine (Sigma-Aldrich), released for 6 hrs, washed to remove the thymidine, and then exposed again to 2.5 mM thymidine this time for 16 hrs in normoxia or hypoxia. The cells were then released from the double thymidine block by culturing in 2% FBS-containing fresh media with or without 2 units/mL of rhEPO and allowed to progress through G1 and into S-phase. The percentage of proliferating cells was determined at 0, 2, 4, 6, 9 and 12 hrs after release from the double thymidine block using the Click-iT® EdU Alexa Fluor® 647 Flow Cytometry Assay Kit (Life Technologies, Carlsbad, CA) according to the manufacturer's instructions. EdU (5-ethynyl-2´-deoxyuridine) is a thymidine analog that becomes incorporated into DNA during active cellular DNA synthesis. Detection is determined via a copper catalyzed covalent reaction between an azide (conjugated to Alexa Fluor 647) and an alkyne. EdU (10 μM) was added to each well 2 hrs prior to harvesting. Cells were trypsinized and fixed in 4% formaldehyde. Cell Quest Pro Software determined cellular DNA synthesis using FlowJo Software. Three independent experiments were performed in triplicate.

### In vivo tumorigenicity

Animal care was in compliance with the recommendations of *The Guide for Care and Use of Laboratory Animals* (National Research Council) and approved by our local IACUC. The subcutaneous tumorigenicity assay was performed in athymic BALB/c (nu/nu) mice, 6 to 8 weeks old purchased from Harlan Laboratories (Indianapolis, IN). Procrit (epoetin α; Amgen Inc, Thousand Oaks, CA) was used for the *in vivo* treatment of EPO. The properties of rhEPO were tested *in vivo* using a subcutaneous xenograft model by inoculating 10^6^ Caki-1, 786-O and 769-P cells as described previously [16,19]. Since RPTEC cells are benign and not known to produce xenograft tumors, this cell line was not tested *in vivo.* After 24 hrs, mice were divided randomly into two groups (control or 200 international units (IU)/kg of rhEPO) and treatment was initiated. RhEPO was administered subcutaneously once weekly. Control mice received vehicle alone (PBS) on the same schedule. At least 10 animals were in each group. Tumor volumes were measured twice weekly with digital calipers and calculated by V (mm^3^) = length × (width)^2^ × 0.5236. After 10 wks of treatment, the mice were sacrificed. However, 30 mins before being sacrificed, each mouse was intraperitoneally injected with 0.1 mL (60 mg/kg of body weight) of pimonidazole hydrochloride (Hypoxyprobe-1 Plus Kit; Hypoxyprobe Inc., Burlington, MA), according to the manufacturer's instructions [20]. Subsequently, the mice were sacrificed and xenografts resected. The excised tumors were placed in 10% buffered formaldehyde solution and embedded in paraffin. Paraffin blocks were sectioned for H&E staining and immunohistochemical (IHC) staining.

### Immunohistochemical (IHC) analysis of xenograft tumors

Paraffin embedded tumors were sectioned (4 μm), deparaffinized in xylene and rehydrated using graded percentages of ethanol. Slides were treated with 1% hydrogen peroxide in methanol to block endogenous peroxidase activity. Staining was conducted using anti-human EPO antibody (sc-7956, dilution 1:200), anti-human EPOR antibody (sc-695, dilution 1:100), HIF-1α (sc-53546, dilution 1:100), VEGF (sc-152, dilution 1:200), cyclin D1 (#2978, dilution 1:50), p21^cip1^ (#2947, dilution 1:100), p27^kip1^ (#3686, dilution 1:200), anti-human Ki-67 (MIB-1, dilution, 1:200; Dako). Biotin-labeled horse anti-mouse IgG or rabbit IgG (2 μg/ml in 1% BSA blocking buffer) was used as secondary antibody. Immunoreactive signals were amplified by formation of avidin-biotin peroxidase complexes and visualized using 3, 3'- diaminobenzidine (DAB). Nuclear counterstaining was conducted with hematoxylin. Proliferative index analysis was determined as previously described [16]. In addition, slides were immunostained with fluorescein isothiocyanate (FITC)–conjugated primary antibody against pimonidazole (1:50) and horseradish peroxidase–labeled secondary anti-FITC monoclonal antibody (1:50) supplied with the hypoxia detection kit (Hypoxyprobe-1 Plus Kit), according to a modification of the manufacturer's instructions as described previously [20].

### Statistical analyses

All data are expressed as mean ± standard deviation (SD) and mean ± standard error of the mean (SEM). Statistical analyses were conducted using GraphPad Prism 5.0 (GraphPad Software, Inc.). The comparison between EPO and EPOR expression in cancer *vs.* benign tissue was calculated using Mann–Whitney U test. For most *in vitro* and *in vivo* comparisons, a 2-tailed unpaired Student t test or Mann–Whitney U test was conducted. Differences were considered statistically significant at *p* < 0.05.

## Results

### Erythropoietin and erythropoietin receptor expression is upregulated in human cancers

**Figure 1:**
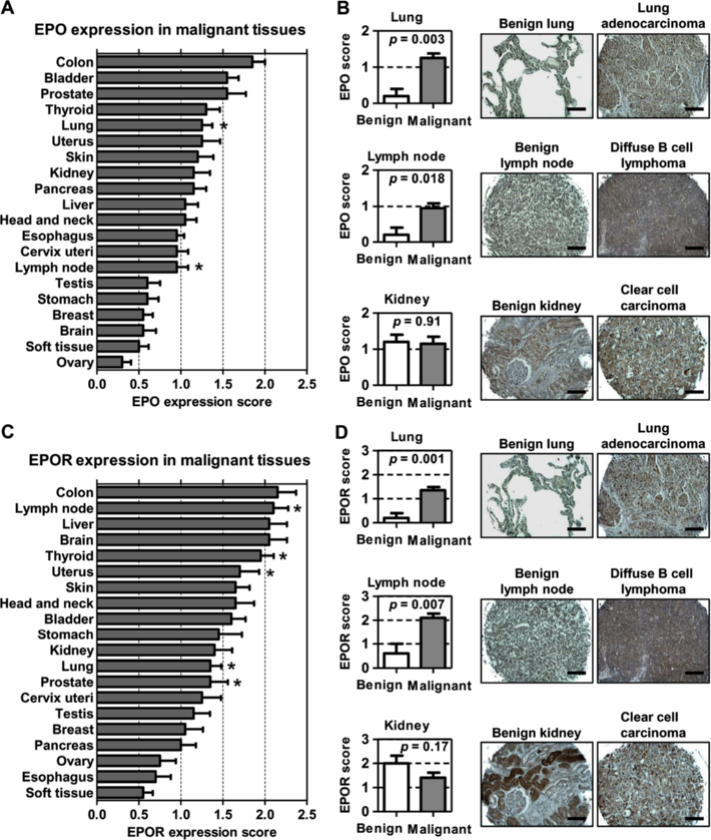
**Erythropoietin (EPO) and erythropoietin receptor (EPOR) expression in human malignancies.** The expression scores of EPO **(A)** and EPOR **(C)** in 20 different malignancies are shown in a bar graph. The expression score (0 to 3) was quantified by combining the proportion and intensity scores. Asterisks indicate malignancies having a significant difference (*p* < 0.05) between malignant tissues and corresponding benign tissue. Representative images of EPO **(B**, right panels**)** and EPOR **(D**, right panels**)** immunostaining of normal lung, lymph node and kidney are illustrated with lung cancer, lymphoma and kidney cancer. Lung cancer and lymphoma, but not renal cell carcinoma, were noted to have an increase expression of EPO **(B**, left panels**)** and EPOR **(D**, left panels**)**.

We analyzed a human cancer TMA consisting of malignant and benign tissue from 20 organ sites. The immunohistochemistry expression scores from cancerous tissue were compared to those of corresponding benign tissue (Figure 1A). The EPO expression score was significantly elevated in lung cancer (*p* = 0.003) and lymphoma (*p* = 0.018). Of note, EPO expression scores in RCC (1.15) and benign renal tissue (1.20) were not significantly different (*p* = 0.91). Figure 1B shows representative images of EPO immunostaining in lung cancer, lymphoma and RCC. We also scored EPOR expression in the TMA specimens (Figure 1C). The EPOR expression score was significantly elevated in lung (*p* = 0.011), lymphoma (*p* = 0.007), thyroid (*p* = 0.032), uterine (*p* = 0.038), and prostate cancers (*p* = 0.011). EPOR expression scores in RCC (1.4) and benign renal tissue (2.0) were not significantly different (*p* = 0.17). Figure 1D shows representative images of EPOR immunostaining in lung cancer, lymphoma and RCC. The lack of EPO or EPOR correlation to RCC substantiates the previous report by Papworth *et al.*[21].

### Exposure of hypoxic human renal cells to recombinant erythropoietin stimulates cellular proliferation

**Figure 2:**
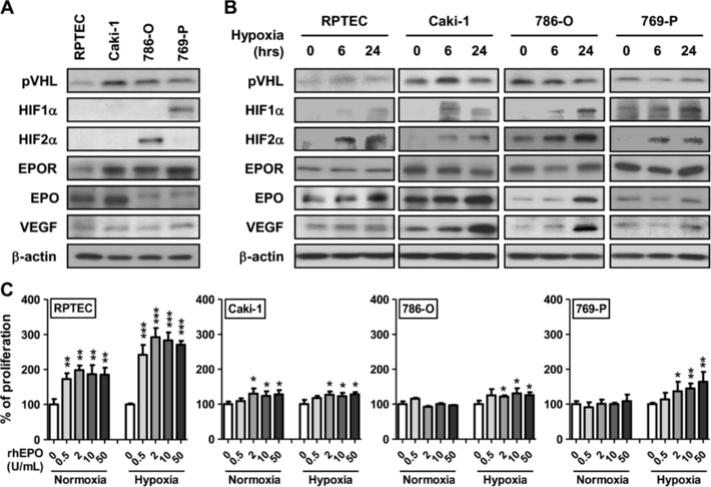
**Effect of recombinant human erythropoietin and hypoxia on the proliferative potential of human renal cell lines. A**, Western blot analysis of four human renal cell lines was done to confirm EPO and EPOR status. Furthermore, other key molecules (*e.g*., VHL, HIF-1α, HIF-2α and VEGF) related to clear cell RCC were noted. Cells were grown in complete media in normoxic condition and total cellular protein lysate in the exponential phase were collected for analysis. **B**, Western blot analysis of four human renal cell lines exposed to hypoxia for 6 and 24 hrs was perform to note any change in the molecular status evident from normoxic conditions. β-actin is used as a loading control. **C**, Proliferation rate was measured in four human renal cell lines cells exposed to normoxia or hypoxia and grown in the indicated doses of recombinant human EPO (0–50 units/mL) at 48 hrs. Data were represented as mean ± SD relative to untreated cells, which are set to 100%. Three independent experiments were performed in triplicate. Significance compared to untreated cells is denoted by *, *p* < 0.05; **, *p* < 0.01, ***, *p* < 0.001.

We next investigated whether rhEPO might influence cellular proliferation in a panel of human renal cell lines. Key molecules associated with clear cell RCC, as well as EPO and EPOR status were determined in a panel of human renal cell lines comprised of RPTEC, Caki-1, 786-O and 769-P (Figure 2A). We know that expression of the EPO gene is regulated by hypoxia through transcriptional regulators family of hypoxia-inducible factors (HIF) [22], so we also assessed the same key molecules in the cell line panel after exposure to hypoxia over the course of 24 hrs. Hypoxia treatment resulted in the increase of HIF-1α, HIF-2α, EPO and VEGF in all cell lines tested (Figure 2B). A slight increase in EPOR expression was noted in 786-O and 769-P cells exposed to hypoxia, but no changes in VHL expression were observed. We then investigated whether exposing human renal cells to increasing doses of rhEPO could affect cellular proliferation. In an *in vitro* proliferation assay at 48 hrs, proliferation of RPTEC and Caki-1 cells was significantly enhanced by exposure to 0.5 units/mL rhEPO (*p* = 0.001) and 2 units/mL rhEPO (*p* = 0.04), respectively, while the cell lines 786-O and 769-P were unaffected, even at the highest concentration of rhEPO (50 units/mL). Parallel *in vitro* proliferation assays under hypoxic conditions were also performed. The observed proliferation of RPTEC and Caki-1 cells was significantly enhanced by the exposure of 0.5 units/mL rhEPO (*p* = 0.0009) and 2 units/mL rhEPO (*p* = 0.03), respectively. Furthermore, in this hypoxic state, the proliferation of 786-O and 769-P was also significantly increased by the addition of 2 units/mL rhEPO (*p* = 0.03 and *p* = 0.04, respectively) (Figure 2C). Thus, in cells with non-functional, mutated VHL (786-O and 769-P) and thus constitutive expression of HIF, rhEPO was able to stimulate cellular proliferation only under hypoxic conditions. Conversely, in cells with functional, wild-type VHL and no HIF expression (RPTEC and Caki-1), rhEPO could stimulate proliferation in both normoxic and hypoxic states.

### Exposure of renal cells to **recombinant erythropoietin** causes progression through G1-phase of the cell cycle by differentially regulating cell cycle proteins

**Figure 3:**
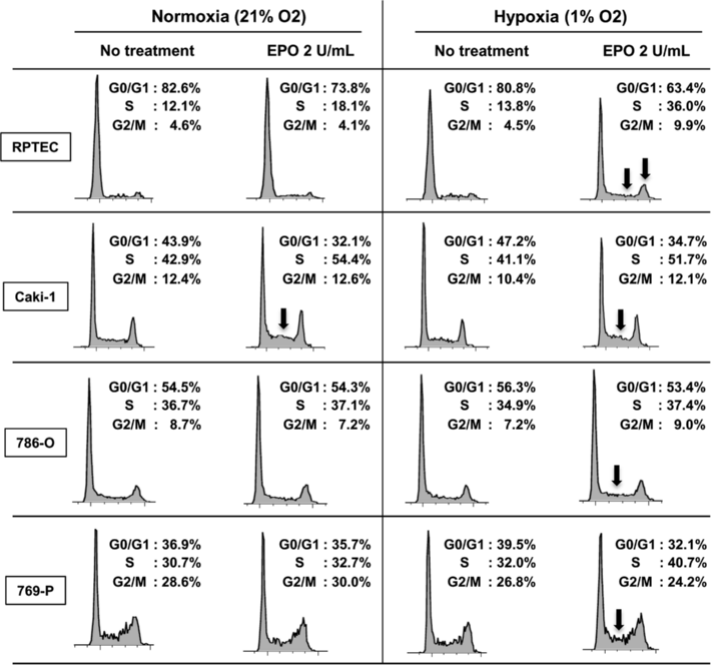
**The effects of erythropoietin on cell cycle.** Cells, which were starved for 18 hrs in normoxic or hypoxic conditions then treated with or without rhEPO for additional 10 hrs in normoxic or hypoxic condition, were analyzed. Specifically, the percentage of population in G_0_/G_1_, S, and G_2_/M phase of the cell cycle were analyzed by flow cytometry after propidium iodide staining of cellular DNA. Arrows indicate the major changes in EPO-treated cells compared to untreated cells. Data are representatives from three independent experiments.

**Figure 4:**
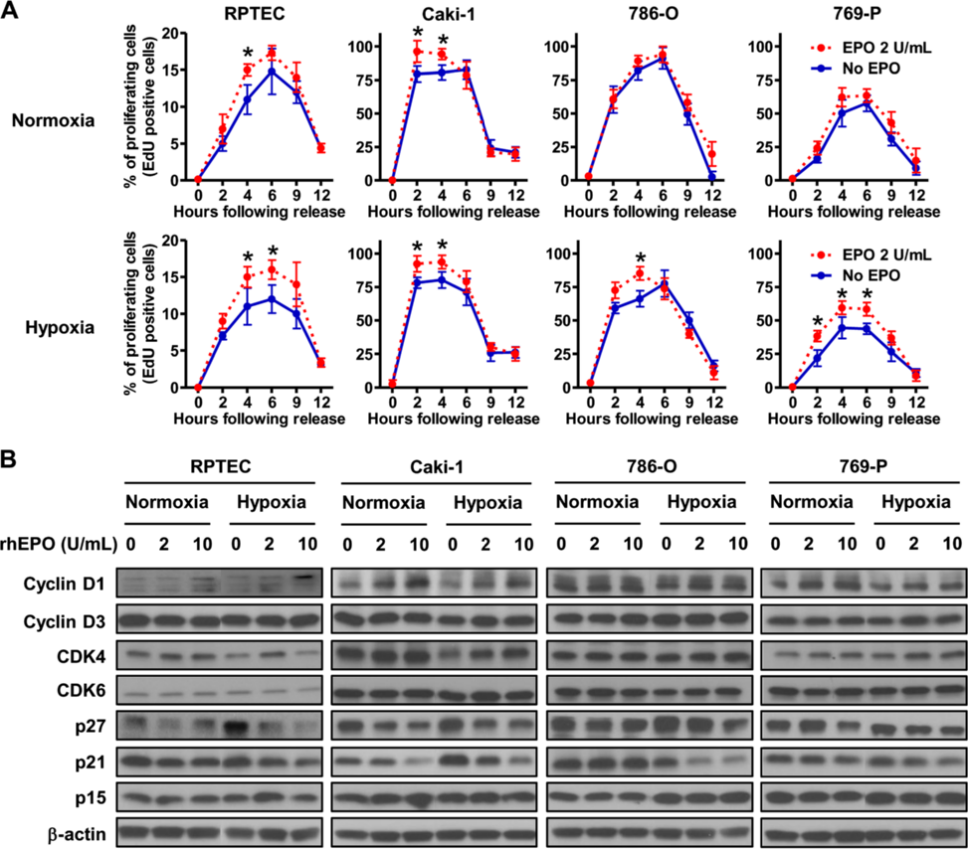
**Erythropoietin promotes S phase progression. A**, Cells were synchronized in G_0_/G_1_-phase by using a double thymidine block and S-phase entry was monitored by the EDU incorporation following thymidine release. The percentage of proliferating cells at the indicated time after release was determined. The result of normoxia and hypoxia are shown in upper panels and lower panels, respectively. Asterisks indicate the significant difference (*p* < 0.05) between untreated cells (solid blue line) and rhEPO-treated cells (dashed red line). Data were represented as mean ± SD from three independent experiments. **B**, Cyclins, cyclin-dependent kinases and cyclin-dependent kinase inhibitors which are known to be keys for G_1_/S transition were analyzed by Western blot to monitor the association of the stimulation of EPO and transduction of cell cycle proteins. Renal cells were treated with the indicated concentrations of rhEPO for 24 hrs in normoxic and hypoxic condition. Cell lysates were subjected to Western blot analysis. β-actin was used as a loading control.

Standard FACS cell cycle analysis of the panel of cell lines treated with and without rhEPO under normoxic and hypoxic conditions revealed only subtle changes (*e.g.,* S-phase accumulation in RPTEC and 769-P cells treated with rhEPO in hypoxia) (Figure 3). Using a double thymidine block protocol that effectively arrested 98% of the cells at the G_0_/G_1_-phase of the cell cycle, we were able to more thoroughly assess whether EPO is required for S-phase progression. Cells were released from the double thymidine block by exposing the cells to 2% FBS-containing media with or without 2 units/mL of rhEPO under normoxia or hypoxia (Figure 4A). Synchronized cells of all cell types were more sensitive to rhEPO under hypoxia compared with normoxia. This was more pronounced in RPTEC and 769-P cells. Thus, exposure to rhEPO in a hypoxic state selectively promotes progression from G1 to S-phase, a phase disproportionately represented in frequently dividing cells such as cancer cells. This is the first mention of this phenomenon in the literature.

The expression of molecules that regulate passage of cells from G_0_/G_1_ to S-phase was analyzed by Western blot (Figure 4B). No significant changes in these molecules were noted in cells exposed to hypoxia, except that p27 ^kip1^ was disproportionately elevated relative to cyclin D1 in RPTEC cells. However, upon stimulation with rhEPO in the hypoxic state, cellular levels of cyclin D1 were increased, while cellular levels of p21^cip1^ and p27^kip1^ were reduced. Conversely, when only rhEPO stimulation was present, only cyclin D1 was increased in RPTEC and Caki-1, and p21^cip1^ and p27 ^kip1^ were decreased in Caki-1 and 769-P. Our data suggests that in the presence of hypoxia, rhEPO stimulates cellular proliferation in renal cells by promoting progression through G1 into S-phase through upregulation of cyclin D1 and reduction of cell cycle inhibitors (p21^Cip1^ and p27^kip1^).

### Identification of MAPK-ERK1/2 pathway as specific signaling downstream of erythropoietin resulting in S-phase progression

**Figure 5:**
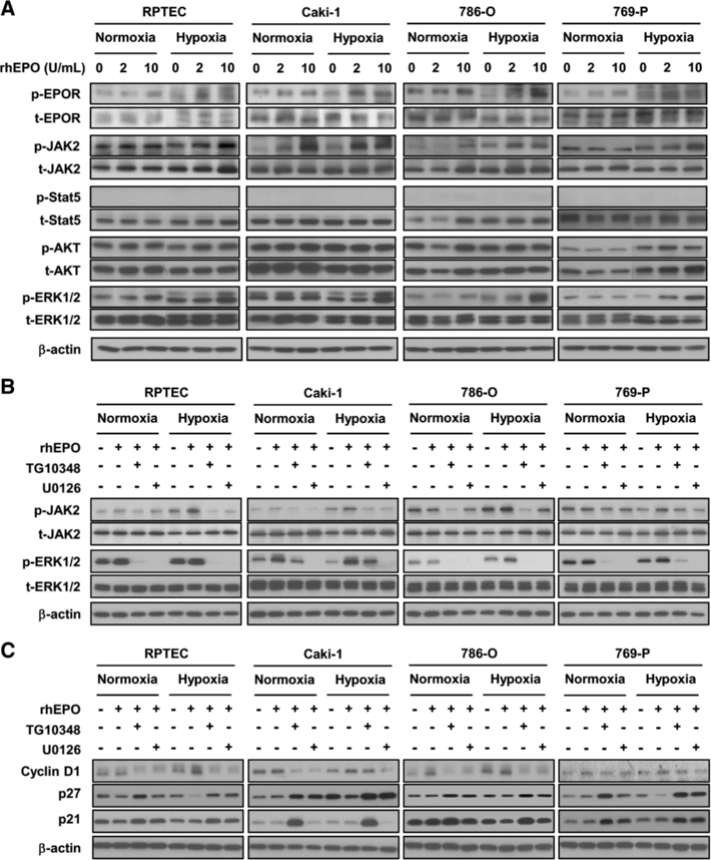
**Erythropoeitin activates the JAK and MAPK/ERK pathways. A**, Four renal cell lines were starved in serum/growth factor-free media containing 0.1% BSA in normoxia or hypoxia. Cells were stimulated with the indicated concentration of rhEPO. Immunoblotting of protein extracts with indicated antibodies (at left of panel) shows JAK2 and MAPK/ERK pathway components in renal cell lines stimulated with rhEPO in the presence of hypoxia. β-actin is used as a loading control. **B**, Four renal cell lines were starved in serum/growth factor-free media containing 0.1% BSA in normoxia or hypoxia. Cells were subjected to 1 μM of TG10348 (a JAK2 inhibitor) or 1 μM of U0126 (a MEC inhibitor) for 60 mins prior to the addition of 10 units/mL rhEPO. Ten minutes after exposure of rhEPO, cell lysates were collected and subjected to Western blot analysis with the indicated antibodies. β-actin is used as a loading control. **C**, Cells are treated with the indicated concentrations of rhEPO in media containing 2% FBS for 24 hrs in normoxic or hypoxic condition. Cell lysates are subjected to Western blot analysis. Western blot analysis shows cyclin D1 was induced and p27 ^kip1^ and p21 ^cip1^ were down-regulated in renal cells stimulated with rhEPO in the presence of hypoxia. β-actin served as loading control.

Previous studies have linked EPO-induced changes to activation of JAK2 and MAPK-ERK1/2 pathways in some model systems [23-26]. To confirm that the proliferative effects of EPO are mediated through the activation of JAK2 and MAPK-ERK1/2 in human renal cells, and to evaluate if these same pathways are involved when cells are subjected to a hypoxic environment, we monitored the expression of JAK2, phosphorylated JAK2 (p-JAK2), Stat5 and phosphorylated Stat5 (p-Stat5) to assess the JAK2 pathway, and Akt, phosphorylated Akt (p-Akt), ERK1/2 and phosphorylated ERK1/2 (p-ERK1/2) to assess the MAPK-ERK1/2 pathway. Under normoxic conditions, exposure to rhEPO resulted in an increase in the expression of p-JAK2 and p-ERK1/2 in RPTEC cells, an increase in p-JAK2 in Caki-1 cells, and an increase in p-JAK2, p-AKT and p-ERK1/2 in 786-O cells. No changes were observed in 769-P cells (Figure 5A). Hypoxic culture alone was associated with an increase in the expression of p-ERK1/2 in RPTEC cells, p-JAK2 in Caki-1 cells, p-JAK2 in 786-O and p-JAK2 and p-Akt in 769-P cells. Most notably, in the hypoxic state, the addition of EPO consistently increased the expression of p-JAK2 and p-ERK1/2 in all four cell lines (Figure 5A). Subsequently, we set out to evaluate which pathway, JAK2 or MAPK-ERK1/2, was involved in the observed molecular changes associated with G1-phase progression. This was achieved by targeting each pathway with a small molecule inhibitor (TG101348 targets JAK2 and U0126 targets MEK in the MAPK-ERK1/2 pathway). In all cell lines, and under all experimental conditions (+/− rhEPO and hypoxia/normoxia), TG101348 treatment resulted in a reduction in p-JAK2, and U0126 treatment resulted in a reduction of p-ERK1/2 (Figure 5B). In parallel experiments utilizing these inhibitors, we assessed changes in cell proliferation (Additional file 1: Figure S1), specifically G1-phase progression by Western blot analysis, which documented changes in cyclin D1, p21^cip1^ and p27^kip1^ expression (Figure 5C). We conclude that EPO exposure results in the activation of both the JAK2 and ERK1/2 pathways leading to changes in proliferation under hypoxic conditions.

### Effects of systemic administration of recombinant erythropoietin in a mouse xenograft tumor model

**Figure 6:**
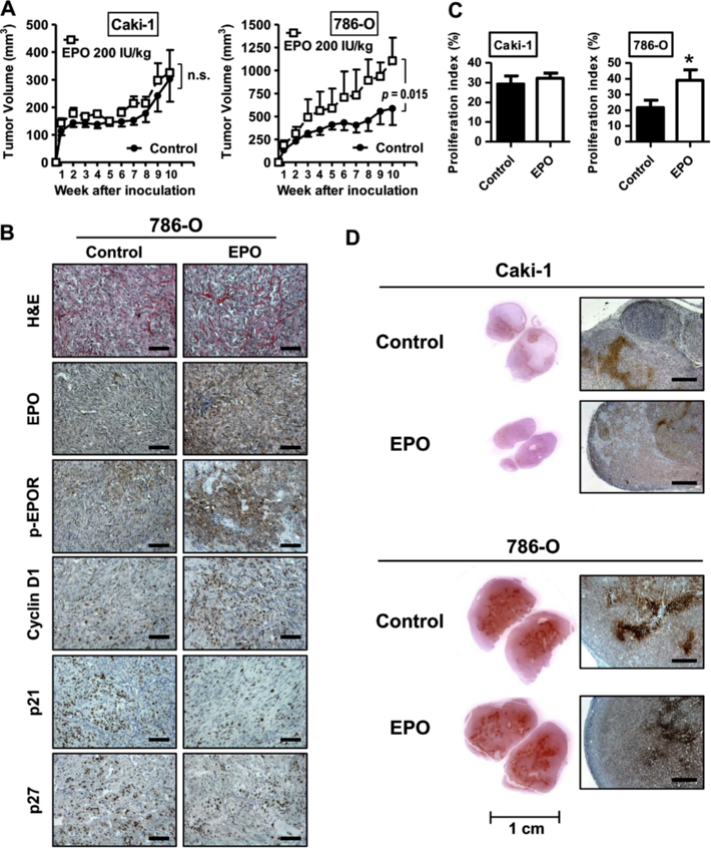
**Erythropoietin increases xenograft tumor growth of human 786-O renal cell carcinoma cells.** Xenograft tumors were established by subcutaneous injection of Caki-1 and 786-O cells into athymic nude mice (nu/nu). One day after cell injection, administration of rhEPO was initiated as described in Material and Methods. **A**, The tumor size was monitored over 10 wks and plotted as mean ± SEM from the two treatment groups per cell line (n = 10 per group). Treatment with rhEPO was associated with an increase in tumor burden among 786-O xenograft tumors. **B**, Representative pictures of 786-O tumors of H&E staining and IHC staining for EPO, phospho-EPOR, cyclin D1, p21^cip1^ and p27^kip1^ are shown. Original magnification, 200 ×. Scale bars, 100 μm. **C**, Proliferative index (%) was quantified based on Ki-67 staining of tumor xenografts of Caki-1 and 786-O. *, *p* < 0.05. **D**, Immunohistochemical localization of pimonidazole hydrochloride (Hypoxyprobe-1) adducts in subcutaneous tumors of Caki-1 and 786-O. Original magnification in right panels, 50 ×. Scale bars, 500 μm.

To determine whether EPO can regulate tumor growth and proliferation *in vivo*, we injected subcutaneously Caki-1, 786-O and 769-P cells in athymic nude mice, however, 769-P cells did not form subcutaneous tumors in this model. Systemic administration of rhEPO over the experimental term of 10 wks resulted in a remarkable increase in 786-O tumor size compared to control. Specifically, at the end of the study, control 786-O xenografts achieved an average volume of 603 mm^3^ compared to 1107 mm^3^ (*p* = 0.015) for 786-O tumors treated with 200 IU/mg/week (Figure 6A). However, administration of EPO in Caki-1 xenografts did not result in a tumor growth advantage compared to controls (*p* = 0.20) (Figure 6A). Evaluation of excised xenografts revealed a clear increase in cyclin D1 and a reduction in p21^cip1^ and p27^kip1^ in EPO-treated 786-O tumors (Figure 6B). Furthermore, an increase in p-EPOR expression was noted in 786-O xenograft tumors compared to 786-O xenograft controls (Figure 6B). Immunostaining of Caki-1 xenograft tumors are depicted in Additional file 2: Figure S2. The proliferative marker, Ki-67, was studied within the tumor sections and an enhanced Ki-67 positivity was noted in EPO-treated 786-O xenograft tumors. No changes in proliferative index were noted in Caki-1 xenografts treated with rhEPO (Figure 6C). Our *in vitro* data suggested that hypoxia potentiates rhEPO proliferative effects. So at the termination of the *in vivo* experiment, pimonidazole staining assessed the extent of xenograft hypoxia. Interestingly, in the Caki-1 xenografts, which had no increase in tumor growth when exposed to rhEPO, limited areas of hypoxia were noted. Conversely, the 786-O xenografts had a considerable number of hypoxic regions (Figure 6D). These *in vivo* observations confirm the potential of EPO to stimulate cellular proliferation and, hence, tumor growth, especially in a hypoxic setting.

## Discussion

Questions were first raised about the possible exacerbating influence of EPO on human tumors after a landmark study was published in 2003 [12]. Specifically, Heinke *et al.* reported significantly shorter progression-free survival and overall survival in a cohort of head and neck cancer patients who were receiving radiation therapy and rhEPO, the latter presumably administered to overcome therapy-induced anemia. In a comparable cohort, Overgaard and colleagues subsequently reported a similar reduction in survival of head and neck patients undergoing tumor therapy while receiving rhEPO [27]. Table 1 illustrates the meta-analysis results of Glaspy *et al.* that examined EPO affects on disease progression in cancer patients receiving chemotherapy [28]. When outcomes were analyzed ‘per protocol’, there was no significant effect of rhEPO on disease progression. However, a post-hoc analysis reported by Henke *et al.* including erythropoietin receptor (EPOR) expression suggested that loco-regional progression-free survival was poorer in patients with EPOR-positive tumors receiving rhEPO [29]. Unfortunately, additional studies using this EPOR antibody revealed problems of non-specific binding of the antibody thus reducing the validity of these results [30]. In the genitourinary literature, only limited reports have commented on RCC disease progression in patients receiving rhEPO [31-33]. Thus, the equivocal data does not allow one to draw definitive conclusions. Consequently, we are confronted with conflicting results when assessing the affects of rhEPO administration in cancer patients.

**Table 1:** Meta-analysis results of oncology trials that examined erythopoietin’s affect on disease progression in patients receiving chemotherapy

**Study publication**	**Tumor type**	**No. of patients analyzed**	**Odds ratio (95%) for disease progression**
Osterborg *et al.* 1996[34]	Hematologic	144	1.20 (0.60-2.40)
Littlewood *et al*. 2001[35]	Solid (non-hematologic)	375	0.64 (0.40-1.02)
Pronzato *et al.* 2010[36]	Breast	223	1.02 (0.46-2.26)
Vansteenkiste *et al.* 2002[37]	SCLC and NSCLC	314	0.58 (0.30-1.11)
Hedenus *et al.* 2003[38]	Hematologic	344	1.08 (0.66-1.76)
Vadhan-Raj *et al.* 2003[39]	Gastric and rectal	60	1.01 (0.35-2.94)
Chang *et al.* 2005[40]	Breast	354	0.82 (0.39-1.72)
Grote *et al.* 2005[41]	SCLC	224	0.85 (0.50-1.44)
Leyland-Jones *et al.* 2005[42]	Breast	939	0.84 (0.64-1.08)
Osterborg *et al.* 2005[43]	Hematologic	343	0.74 (0.44-1.25)
Witzig *et al.* 2005[44]	Mixed	344	1.20 (0.75-1.91)
Wilkinson *et al.* 2006[11]	Ovarian	181	7.47 (0.95-58.54)
Engert *et al.* 2007[45]	Hodgkin’s lymphoma	1303	0.86 (0.33-2.24)
Aapro *et al.* 2008[46]	Breast	463	1.07 (0.82-1.40)
Pirker *et al.* 2008[47]	SCLC	596	0.87 (0.52-1.46)
Strauss *et al.* 2008[48]	Cervical	74	0.87 (0.32-2.33)
Thomas *et al.* 2008[49]	Cervical	109	1.02 (0.48-2.15)

Similarly, *in vivo* model studies on the topic are contradictory. In a Lewis lung carcinoma xenograft model, rhEPO was noted to increase primary tumor growth [50]. However in ovarian and other xenograft models, systemic administration of rhEPO did not result in growth of primary tumors [51,52]. Our results demonstrate the importance of assessing more than one cell line *in vitro* and *in vivo*. Though all of the cells in our study possessed EPOR, we demonstrated that the administration of rhEPO resulted in the stimulation of growth of 786-O xenograft tumors, but not of Caki-1 xenografts. The only significant difference in the composition of these xenograft tumors was that 786-O possessed more regions of hypoxia; a state in which significantly exacerbates the effects of rhEPO *in vitro*. It was critical to assess these cell lines in an *in vivo* model, because similar to Fujisue and others [53], we noted in *in vitro* that Caki-1 cells had an increase in proliferation when exposed to rhEPO in the normoxic or the hypoxic state. However, this was not reproduce in the xenograft model thus we were able to postulate that tumors with a reduced oxygen tension (*e.g.,* large, expansive tumors) are more likely to be stimulated when exposed to EPO. Regarding our *in vivo* experiments, we noted a failure of 769-P cells to grow as subcutaneous tumors in nude mice. Though reported as tumorigenic by ATCC, limited studies have reported on this aspect [54,55]. However, our *in vitro* results of 769-P cells are similar to previously published 769-P *in vitro* results [53].

In our IHC tissue arrays in which tissue hypoxic status was unknown, EPO expression score was significantly elevated in lung cancer (*p* = 0.003) and lymphoma (*p* = 0.018), but not in RCC (*p* = 0.91). Furthermore, EPOR expression score was significantly elevated in lung (*p* = 0.011), lymphoma (*p* = 0.007), thyroid (*p* = 0.032), uterine (*p* = 0.038) and prostate cancers (*p* = 0.011), however it was not elevated in RCC (*p* = 0.17). The lack of EPO or EPOR correlation by IHC in RCC *vs.* benign samples substantiates a previous large cohort (n = 195) reported by Papworth *et al.*[21], but is contradictory to two small studies from Asia (combine n = 129) [56,57]. Interestingly a recent study noted that EPO levels were elevated in high stage RCC compared to low stage RCC [58]. Thus further investigation into this, and correlating the tumor hypoxic status to EPO/EPOR expression may be warranted.

Our results provide evidence that EPO exposure leads to stimulation of JAK2 and ERK1/2 signaling, which in turn positively regulates progression through the cell cycle by inducing cyclin D1 and inhibiting p21^cip1^ and p27^kip1^ expression (Figure 4). The progression through the cell cycle is further potentiated under hypoxic conditions. Tumor hypoxia is noted in approximately 30% of RCC [59] and is known to increase in all lesions as tumor burden increases. In this study, we present clear evidence that rhEPO is a potent mitogen, especially under hypoxia. Through pharmacologic stimulation, we also show that active JAK2 and ERK1/2 signaling tightly controls cyclin D1 expression in a panel of human cell lines (Figure 5). We have also found that exposure to rhEPO resulted in significant growth of 786-O xenografts (which contained many regions of hypoxia), with concomitant increased expression of cyclin D1 (Figure 6).

It is known that active EPOR can stimulate JAK2 kinase [23] and cause subsequent activation of multiple signaling pathways, including the MAPK-ERK-1/2 pathway [24]. For example, Jeong *et al.* treated human ovarian cells with rhEPO (50,000 mU/ml) and noted an increase in the phosphorylation of extracellular signal related kinase (ERK)-1/2, but no change in cellular growth or survival [25]. Similarly, treatment of lung cancer cells resulted in an increase in ERK-1/2 levels [50]. We were able to confirm that rhEPO can induce JAK2 and ERK1/2 expression in renal cell lines. Furthermore, the increase in cellular proliferation seen with rhEPO could be abrogated with the addition of the JAK2 or ERK1/2 inhibitor (Additional file 1: Figure S1). Thus, cells can circumvent JAK2-dependent pathway for the JAK2-independent pathway (ERK1/2). Mannello and other previously reported about a JAK2-independent pathway [60].

After synchronizing cells with a double thymidine block strategy, exposure to rhEPO was noted to more rapidly advance the cells through the cell cycle. Cursory studies have described how EPO may affect molecules related to cell cycle. For example, STAT5 is an intracellular protein associated with the cytoplasmic portion of EPOR with a noted interplay between the phosphorylation of JAK2 and STAT5 [61]. Phosphorylated JAK2 forms homodimers and translocates to the nucleus where it directly binds to the DNA and activates cyclin D1 [22]. We showed that EPO stimulation of two renal cell lines, RPTEC (normal primary human renal tubule epithelial cells with wild-type VHL) and Caki-1 (clear cell RCC with wild-type VHL), under normoxic conditions resulted in cyclin D1 overexpression. But in hypoxic conditions, rhEPO stimulation resulted in cyclin D1 upregulation in all four renal cell lines tested (Figure 3D), and this induction was accompanied by unabated progression through G1-phase of the cell cycle. Furthermore, rhEPO treatment, both in normoxic and hypoxic conditions, resulted in a down regulation of p21^cip1^ and p27^kip1^. Downregulation of these molecules was more pronounced during hypoxia, shedding light on molecular mechanisms involved and further confirming that EPO effects are exacerbated by hypoxia. The re-evaluation of large cohorts with respect to EPO and hypoxic state of the tumor could shed light on this phenomenon and help direct future clinical trials. These data presented herein suggest that rhEPO treatment may have adverse effects in specific scenarios and thus the use of rhEPO in the cancer patient should be considered carefully weighing the benefits and risks.

## Abbreviations

EPO: Erythropoietin; TMA: Tissue microarray; rhEPO: Recombinant erythropoietin; HIF: Hypoxia inducible factor; RCC: Renal cell carcinoma; VHL: von Hippel-Lindau; EPOR: Erythropoietin receptor; BSA: Bovine serum albumin; PI: Propidium iodide; IACUC: Institutional animal care and use committee; IU: International units; IHC: Immunohitochemical staining; FITC: Fluorescein isothiocyanate; DAB: Diaminobenzidine; SD: Standard deviation; SEM: Standard error of the mean.

## Competing interests

Makito Miyake, Adrienne Lawton, Ge Zhang and Evan Gomes Giacoia declare that they have no competing interests, while S. Goodison and C.J. Rosser are officers of Nonagen Bioscience Corporation.

## Authors’ contributions

MM carried out in vitro and in vivo experiments, performed and analyzed IHC. AL analyzed IHC. GZ assisted in in vitro experiments. EGG assisted in in vivo experiments. SG assisted with drafting/revising manuscript. CJR conceived of the study, and participated in its design and coordination and helped to draft the manuscript and secured funding. All authors read and approved the final manuscript.

## Supplementary Material

Additional file 1: Figure S1. Blockade of JAK2 and ERK1/2 by specific inhibitors suppress cellular response to EPO. Cells (10^3^ cells/well) were seeded in 96 well dishes and incubated in normoxic condition. TG10348 (1 μM) or U0126 (1 μM) were added 60 mins prior to the addition of 2 units/mL of rhEPO. The plates were exposed to normoxic or hypoxic conditions. Cell viability was determined at 48 hrs after the exposure to EPO. Data were represented as mean ± SD relative to untreated cells, which are set to 100%. Three independent experiments were performed in triplicate. Significance compared to untreated cells is denoted by *, *p* < 0.05; **, *p* < 0.01, ***, *p* < 0.001. Click here for file

Additional file 2: Figure S2. Representative pictures of Caki-1 xenograft tumors of H&E staining and IHC staining for EPO, phospho-EPOR, cyclin D_1_, p21^cip1^ and p27^kip1^. Original magnification, 200 ×. Scale bars, 100 μm. Click here for file
