# Supplementary figures and images for: Erythropoietin is a JAK2 and ERK1/2 effector that can promote renal tumor cell proliferation under hypoxic conditions
Source: J Hematol Oncol. 2013 Sep 3;6:65. doi: 10.1186/1756-8722-6-65 (PMC3844377; doi:10.1186/1756-8722-6-65)

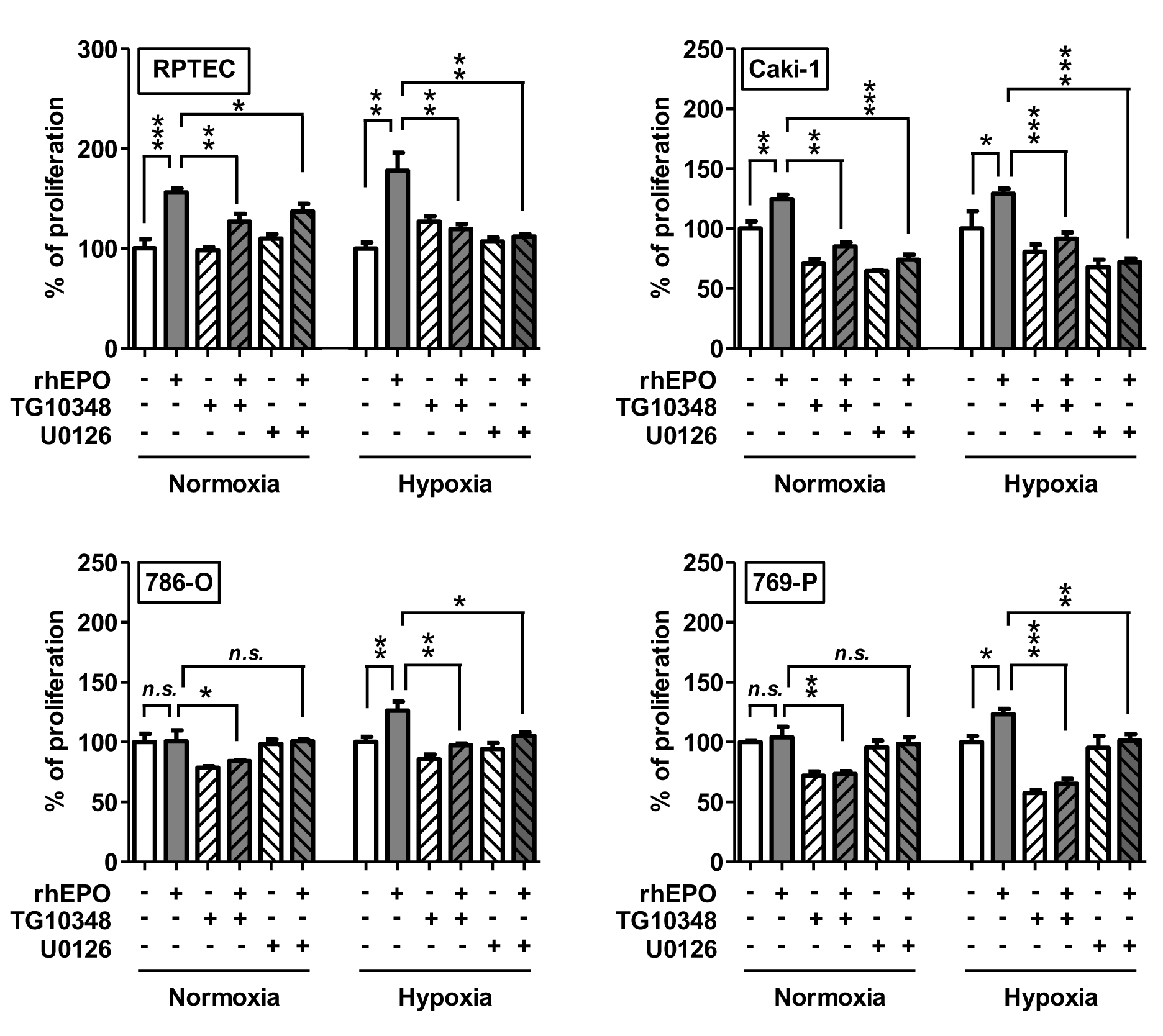

Supplement: Additional file 1: Figure S1. — Blockade of JAK2 and ERK1/2 by specific inhibitors suppress cellular response to EPO. Cells (103 cells/well) were seeded in 96 well dishes and incubated in normoxic condition. TG10348 (1 μM) or U0126 (1 μM) were added 60 mins prior to the addition of 2 units/mL of rhEPO. The plates were exposed to normoxic or hypoxic conditions. Cell viability was determined at 48 hrs after the exposure to EPO. Data were represented as mean ± SD relative to untreated cells, which are set to 100%. Three independent experiments were performed in triplicate. Significance compared to untreated cells is denoted by *, p < 0.05; **, p < 0.01, ***, p < 0.001. [file 1756-8722-6-65-S1.tiff]

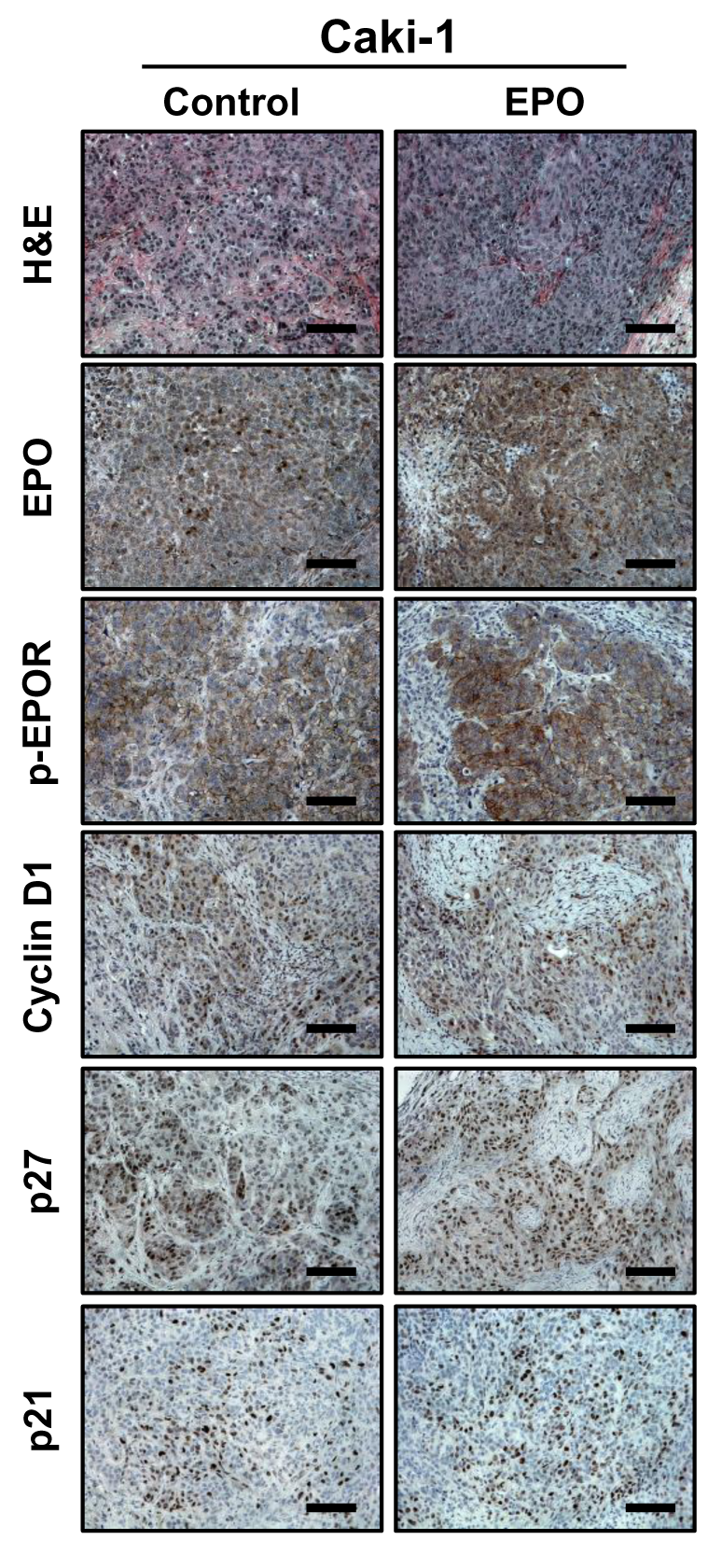

Supplement: Additional file 2: Figure S2. — Representative pictures of Caki-1 xenograft tumors of H&E staining and IHC staining for EPO, phospho-EPOR, cyclin D1, p21cip1 and p27kip1. Original magnification, 200 ×. Scale bars, 100 μm. [file 1756-8722-6-65-S2.tiff]
